# What Kind of Information About Marginal Donors Is Available Through Sources Other Than Health Care Professionals for Patients on the Waiting List for Organ Transplantation?

**DOI:** 10.2196/ijmr.4301

**Published:** 2015-07-14

**Authors:** Sara Kamran, Yvon Calmus, Marie Pascale Pomey, Gwenaëlle Vidal-Trécan

**Affiliations:** ^1^ Paris center university hospitals Public Health ward: Quality and Risk Management Assistance Publique - Hôpitaux de Paris Paris France; ^2^ Doctoral School of Public Health 420 Paris France; ^3^ Pitié Salpêtrière hospital Centre de transplantation hépatique Assistance Publique - Hôpitaux de Paris Paris France; ^4^ Institut de Recherche en Santé Publique Department of Health Administration Université de Montréal Montreal, QC Canada; ^5^ Paris Descartes University Department of Public Health, Medical School Paris France; ^6^ National Institute of Health and Medical Research METHODS research team (1153 unit) Paris France

**Keywords:** access to information, marginal donor, online health information, health information exchange, organ transplantation, lung, liver, kidney, heart

## Abstract

**Background:**

The current organ shortage has necessitated expanding the criteria for potential donations to marginal donors (older or sick donors whose organs would have been considered unsuitable before). In France, physicians are not required to provide information to recipients about marginal donors except for hepatitis C or hepatitis B infection and non-heart-beating donations. We hypothesized that patients can be informed about these risks by other information sources than health care professionals, such as websites and patient associations.

**Objective:**

The objectives of the study were to identify the main health information sources of transplant patients other than health professionals and to evaluate the information provided by websites and associations to patients about the risks of transplantation from marginal donors.

**Methods:**

In this study, the information sources for kidney, liver, heart, and lung patients that had already received transplants or registered on waiting lists were identified by a survey in four transplant centers. Further, the information proposed by French and English language websites and patient associations were evaluated, respectively, by a systematic review of websites and a survey among the presidents of kidney, liver, heart, and lung patient associations.

**Results:**

For the first survey, (367/402) 91.3% responses were registered. Apart from health professionals identified as the principal information source (363/367) 98.9%, 19 liver and 28 heart patients searched for information on the websites, while 37 kidney and 42 lung patients were more informed by patients’ associations. Our two last surveys showed that information about marginal donors is accessible by websites and (10/34) 30% of associations. All of the 60 Internet documents evaluated on French language and English language websites proposed information about marginal donors. Otherwise, (52/65) 80% of these documents were dedicated to health professionals and contained specialized information, difficult to understand by patients. Certain associations, (20/34) 59%, provided information about the risks of transplantation. There were 45/115 patients considering associations as their main information source that were informed by an association’s website. However, only (5/22) 23% of associations communicated the risks of transplantation with patients through their websites.

**Conclusions:**

Currently, patients want to be more informed by other information sources than health professionals, particularly by the websites. Nevertheless, they cannot always trust information proposed by these sources. They need to have their physicians inform them about specialized keywords and present them with reliable information sources. So reliable centers such as universities, transplant centers, and associations should develop the quality and quantity of information proposed to patients on their websites.

## Introduction

### Expanded Criteria Donors

The current organ shortage has necessitated expanding the criteria for potential donation to donors whose organs would have been considered unsuitable before. Kidneys of marginal donors had been used for transplantation in France since 1998 [1] (French national agency of transplantation, ABM’s, report). These donors are termed “marginal” donors, also referred to as "expanded" or "extended" criteria donors. However, these concepts are not clearly defined. “Expanded criteria” is the term most intended for kidney donors. The United Network for Organ Sharing (UNOS) [2] first described these criteria in the United States. They include age 60 or older, or between 50 and 59 with at least two of the following conditions: history of hypertension, creatinine level greater than 1.5 milligram/deciliter, and death caused by a cerebrovascular accident.

The results of our research on Google AdWords in July 2013 showed that the French people did not search some specialized keywords such as "marginal donor" or "expanded criteria donor" on the Google search engine, whereas they searched, on average, 590 times per month the keywords "risks of transplantation" [3]. This means that they want to know more about the risks of transplantation, but that they do not know the specialized keywords. In contrast, people of the United States searched, on average, 170 times per month the specialized keywords “expanded criteria donor" [3]. To find out the reasons of this difference, the main health information sources for patients, and information proposed by them, should be identified.

We thought that patients registered on the French national waiting list (NWL) might not be always informed about the risks and benefits associated with the transplantation of organs from marginal donors by the health care professionals in charge of their care. This hypothesis was supported by the results of a study in submission process that we performed among physicians responsible for transplant centers about the information proposed to patients concerning the risks and benefits associated with the transplantation of organs from marginal donors.

The current regulation makes it mandatory that physicians inform the potential recipient about a donor with a history of hepatitis C or hepatitis B infection [4], or a non-heart-beating donor, especially for a liver or kidney transplantation [5]. Additionally, in France, a law enacted in March 2002 [6] requires that patients be informed about every risk that might occur during a medical procedure. However, no laws, regulations, or instructions force physicians to provide information to recipients about the risks and benefits of organs available from other types of marginal donors.

### Information Proposed to Patients About the Risks and Benefits of Marginal Donors

We only found two studies [7,8] focusing on information proposed to potential recipients about the risks and benefits associated with marginal donors. The authors supported the idea that patients should receive information and may take part in the decision‐making process of whether or not to be transplanted with organs available from a marginal donor.

Adult patients could search for information sources other than health care professionals. According to the results of the “Health Online 2013” survey, realized by the Pew research center [9], adults from the United States got information, care, or support from: a doctor or another health care professional (70%), friends and family (60%), and other patients having the same health conditions (24%). During the past year, certain adults (35%) said that they have searched using the Internet for their or others’ diagnoses. In France, the survey conducted for the National Council of the College of Physicians in 2010 [10] found that the main French health information sources were health care professionals (89%), Internet (64%), relatives (64%), and pharmacists of retail pharmacies (63%).

The hypothesis underlying this study was that apart from health care professionals, Internet websites and patient associations could be two main information sources for transplant patients. Indeed, using the Internet to find information became a current practice, particularly among young people. Furthermore, patients with chronic diseases such as those leading to transplantation often gather in patient associations.

Therefore, the objectives of this study were to: (1) identify the main health information sources of transplant patients; (2) evaluate the use of information sources such as websites or patient associations by patients who had already received transplants or were on the NWL; (3) examine the information provided by patient associations and websites about the risks of transplantation from marginal donors; and (4) compare the information provided by websites in the French and English languages.

## Methods

### Transplant Patients

This study focuses on transplant patients. There were four main organs in terms of number of transplantation (ie, kidney, liver, heart, and lung) [11] that were considered. Through three surveys, we tried to evaluate the information proposed to patients by information sources other than health care professionals about the risks of transplantations from marginal donors.

### First Survey

#### Design

A cross-sectional survey was carried out in July, September, and October 2014, in four transplant centers. During 10 days, all the eligible patients of each center were asked by an anonymous self-questionnaire about their main health information sources.

#### Setting

The four transplant centers were located in the Paris area in different hospitals. A center was chosen for each type of organ (ie, kidney, liver, heart, and lung).

#### Population

Every patient older than 18 years registered on the NWL, or had already received transplants at the age of 18 or above, attending the outpatient consultation of one of the four transplant centers was asked to answer the questionnaire during the study period. Patients attending for reasons other than transplantation, not yet registered on the NWL, canceled their appointment, and the foreign patients who did not know French, were excluded.

#### Data Collection

All patients presenting in transplant centers were given the self-administered questionnaire by an investigator. The investigator was trained for helping patients to fill out the questionnaire, if necessary. She also recorded the number of unfilled out questionnaires and the reason for not filling out the questionnaire (eg, refusal, lack of time).

### Second Survey

#### Design

A systematic review of the information available on websites about the risk factors associated with marginal donors was conducted. The review protocol identified three keywords in French and in English. The search was performed using the Google search engine.

#### Keywords

Keywords were chosen in French and in English. Indeed, first, we found little information on French language websites about the risks associated with marginal donors. Second, some French people may search for information on English language websites. Third, we wanted to compare the kind of information given in French and in English. Fourth, the concept of "Expanded Criteria Donor" was first defined by the UNOS in the United States.

The keywords used were drawn from the specialized vocabulary of health care professionals: «greffon marginal», «donneurs à critère élargis», or «donneur décédé suite à un arrêt cardiaque» in French, and «marginal donor», «expanded criteria donor», or «non-heart-beating donor» in English.

#### Data Collection

According to a literature review about patient Web users [12], patients primarily use a search engine (60% to 92% of patients) to search for health information. We conducted this survey using the Google search engine because it is the most used (78%) in the world [13]. The American Online Advertising Network of CHITIKA [14] reported that websites listed on the first page of Google results generated 92% of all traffic from an average search. Therefore, we decided to review only the first page of Google search results (ten websites) for every keyword.

#### Definition of Variables

To classify the documents, we defined variables according to: (1) the age of document (ie, published before or after 2009), (2) language (ie, French or English), (3) target population (ie, patients or health care professionals), (4) type of information (ie, general or specialized), and (5) accessibility of document (ie, for free or for purchase).

#### Content Analysis

Analyzing the content of the selected documents allowed the pulling out of seven recurrent themes,

1. Definition of marginal donor including classifications for marginal donors, differences between expanded and standard criteria donors, risk factors of marginality, and categories of marginal donors and definition of donor quality score;
2. Results of transplantation from marginal donors including risks, benefits, and statistics associated with the transplantation of organs from various types of marginal donors, and factors influencing the result of the transplantation;
3. Situation of organ shortage and use of marginal donors as a solution including strategies for expanding the organ donor pool, solutions for organ shortage, history of transplantation from marginal donors, and policies for allocation of organs from marginal donors;
4. Process of marginal graft transplantation including decision process, donor selection criteria, characteristics of patients accepting marginal graft, and evaluation of patients’ opinion;
5. Marginal graft optimization;
6. Ethical aspects in transplantation of organs from marginal donors; and
7. Other aspects including surgical techniques of transplantation from marginal donors, cost of transplantation from marginal donors, and guidelines for transplantation from marginal donors.

### Third Survey

#### Survey Design

A cross-sectional survey using an anonymous electronic self-questionnaire was carried out from October 2013 to March 2014 among the presidents of kidney, liver, heart, and lung patient associations to examine the information proposed by these associations to patients about the risks associated with marginal donors. The link of the electronic questionnaire was sent to the presidents of associations in partnership with the ABM. The ABM is recognized as the medical, scientific, and ethical authority in the field of harvesting and transplant of organs, tissues, and cells in France.

#### Population and Setting

The included patient associations were, first, three national federations of kidney, liver, and heart-lung in Paris that federate regional associations (24 for kidney, 11 for liver, and 9 for heart-lung), and then independent associations (3 kidney associations and 1 association for cystic fibrosis). Regional associations were also questioned because their attitude regarding their activities can differ from the national attitude for cultural reasons.

The objectives of these associations are to inform and support patients and their families in the treatment process before or after transplantation, and help them to improve their quality of life.

The associations supporting only tissue or organ donation, or not receiving transplant patients, were excluded.

#### Data Collection

The questions were selected based on relevance to our study questions: “Which health information about risks related to transplantation from marginal donors are proposed by associations?”, and “How could patients receive this information?”. Before deployment, the presidents of three principal French federations of kidney, liver, and heart-lung associations reviewed the questions and were asked to give feedback on whether the questions were understandable for presidents of associations, and whether any questions seemed out of place. Their feedback was incorporated into the survey by 2 of the researchers.

To inform presidents about the context, the purpose, the length of time for the survey, the name of sponsors, and contact information, a leaflet was prepared.

The link of our electronic anonymous questionnaire on the “Survey Monkey” website and an information leaflet were sent by email to the presidents of patient associations. The questionnaire was posted on one page with 10 questions on the website of “Survey Monkey” that captured all of the responses.

The respondents were able to review and change their answers before final validation. Before access to the questionnaire, the Internet Protocol address (numerical label assigned to each computer) was verified by “Survey Monkey”, and a visitor could not respond twice to our questionnaire.

### Statistical Analysis

For all three studies, standard descriptive statistics were performed as appropriate.

In the first survey, a bivariate analysis was performed to identify differences between the information sources of patients in kidney, liver, heart, and lung transplantation. To analyze the answers to multiple choice questions, we chose to consider the distribution of the answers rather than the distribution of the patients.

In the second survey, a bivariate analysis was conducted between the five variables defined previously to search for contrasts. The documents appearing on two websites or dedicated to two organs were considered as two separate documents and counted twice.

Data were compared using chi-square test and Fisher’s exact test as appropriate. The level of statistical significance was set at *P*<.05 using SPSS statistics software version 17.0.0.

In the first and third surveys, the incomplete questionnaires were also analyzed. The percentage was calculated based on the number of answers for each question, but not the number of respondents of the survey.

## Results

### First Survey

During 40 days of survey in four centers, a total of 402 patients were included. There were 367/402 patients (91.3%) that agreed to participate. They were divided into 112/367 women (30.5%) and 255/367 men (69.5%). There were 118/367 kidney (32.1%), 87/367 lung (23.7%), 85/367 liver (23.2%), and 77/367 heart (21.0%) patients that responded to our questionnaire. There were 338/367 patients (92.0%) that had already received transplants.

The main information sources were physicians and health care professionals for (363/367) 98.9%, websites in the French language for (115/367) 31.3%, and patient associations for (105/367) 28.6% of transplant patients. Among health care professionals, (338/363) 93.1% of patients were informed by physicians, (154/363) 42.4% by coordinators, (143/363) 39.4% by nurses of transplant centers, and (95/363) 26.1% by their general practitioners.

Out of 115 patients using “French websites” as an information source, 99 (86.1%), 45 (39.1%), 24 (20.9%), and 24 (20.9%) patients searched for information, respectively, on the Google search engine, websites of patients associations, transplant centers, and the ABM.

Out of 95 patients indicating the keywords most used in their research on Google, 57 (60%) and 45 (47%) patients wrote respectively “kidney OR liver OR heart OR lung AND transplantation” and “kidney OR liver OR heart OR lung AND graft”.

Out of 105 patients informed by associations, 77 (73.3%) used the association’s written documents and 42 (40.0%) consulted the websites or discussion forums organized by the associations. There were 49/105 patients that were members of an association (46.7%). There were (32/49) 65% of these patients that became association members before their transplantation.

There were 51/367 patients (13.9%) that have already participated in a therapeutic education program.

Excluding lung patients, (47/280) 16.8% of patients had heard about marginal graft by their physicians (29/47, 62%), websites (7/47, 15%), and patients transplanted (6/47, 13%). Patients recently transplanted (≥2010) were not more informed about marginal donors than patients transplanted before 2010 (*P*=.994).

Among lung patients, (46/87) 52.9% were suffering from cystic fibrosis.

Mostly heart (29/51) and lung (15/51) patients were participating in the therapeutic education programs. There were 29/51 patients that stated that the therapeutic education programs in which they participated (57%) were organized by transplant centers.

Apart from health professionals identified as the principal information source (Table 1), liver and heart patients searched for information on the websites, while kidney and lung patients were more informed by patients’ associations.

The distribution of health information sources, information provided by physicians or health care professionals, and websites were different according to the organ type (Tables 1-3).

**Table 1:** Distribution of information sources for patients of each type of transplantation (*P*<.001).

Information sources	Kidney, n^a^=200	Liver, n^a^=124	Heart, n^a^=132	Lung, n^a^=199
	n (%)	n (%)	n (%)	n (%)
Physicians or other health care professionals	117 (58.5)	84 (67.8)	75 (56.8)	87 (43.7)
Websites in the French language	32 (16.0)	19 (15.3)	28 (21.2)	36 (18.1)
Patient association	37 (18.5)	10 (8.1)	16 (12.1)	42 (21.1)
Websites in the English language	6 (3.0)	5 (4.0)	5 (3.8)	5 (2.5)
Other patients	4 (2.0)	2 (1.6)	2 (1.5)	11 (5.5)
Other	4 (2.0)	4 (3.2)	6 (4.6)	18 (9.1)

^a^“n” represents the number of responses for each transplant center

**Table 2:** Distribution of information sources provided by physicians or other health care professionals for patients of each type of transplantation (*P*<.001).

Physicians or other health care professionals	Kidney, n^a^=227	Liver, n^a^=185	Heart, n^a^=183	Lung, n^a^=295
	n (%)	n (%)	n (%)	n (%)
Physician of transplant center	112 (49.3)	78 (42.2)	62 (33.9)	86 (29.2)
Nurse coordinator of transplant center	32 (14.1)	37 (20.0)	20 (10.9)	65 (22.0)
Nurses of transplant center	32 (14.1)	28 (15.1)	39 (21.3)	44 (14.9)
General practitioners	27 (11.9)	22 (11.9)	30 (16.4)	16 (5.4)
Psychologist of transplant center	2 (0.9)	9 (4.9)	14 (7.7)	49 (16.6)
Relatives or family member as health care professionals	9 (4.0)	8 (4.3)	9 (4.9)	15 (5.1)
Other physician specialized	13 (5.7)	2 (1.1)	7 (3.8)	12 (4.1)
Other	0 (0.0)	1 (0.5)	2 (1.1)	8 (2.7)

^a^“n” represents the number of responses for each transplant center

**Table 3:** Distribution of information sources provided by websites (French or English) for patients of each type of transplantation (*P*=.02).

Websites (French and English)	Kidney, n^a^=60	Liver, n^a^=33	Heart, n^a^=57	Lung, n^a^=73
	n (%)	n (%)	n (%)	n (%)
Research engine of Google	26 (43)	18 (55)	26 (46)	29 (40)
Websites of transplant association	17 (28)	4 (12)	10 (17)	14 (19)
ABM	7 (12)	6 (18)	4 (7)	7 (10)
Transplant center	5 (8)	1 (3)	12 (21)	6 (8)
Pages of transplant groups in social network	5 (8)	4 (12)	4 (7)	10 (14)
Other	0 (0)	0 (0)	1 (2)	7 (10)

^a^“n” represents the number of responses for each transplant center

**Table 4:** Distribution of information sources provided by associations for patients of each type of transplantation (*P*=.50).

Patients’ associations	Kidney, n^a^=59	Liver, n^a^=16	Heart, n^a^=23	Lung, n^a^=88
	n (%)	n (%)	n (%)	n (%)
Written communication	25 (42)	7 (44)	10 (44)	35 (40)
websites or discussion forum of association	14 (24)	1 (6)	5 (22)	22 (25)
Information session or educational program	8 (14)	1 (6)	2 (9)	6 (7)
Focus group of patients	2 (3)	1 (6)	3 (13)	9 (10)
Annual meeting of members	4 (7)	1 (6)	1 (4)	9 (10)
Question and answer session with a physician	3 (5)	1 (6)	1 (4)	3 (3)
Visit of inpatients by association's members in hospital	2 (3)	2 (13)	1 (4)	3 (3)
Other	1 (2)	2 (13)	0 (0)	1 (1)

^a^“n” represents the number of responses for each transplant center

### Second Survey

In total, sixty documents were found on the first pages of the Google search using each keyword (30 written in French and 30 in English). There were five documents that were dedicated to both kidney and liver, so the total analyzed was 65.

There were (32/65) 49% and (13/65) 20% of documents that were dedicated, respectively, to kidney and to liver transplantation. There were two documents of 65 that were dedicated to lung transplantation, but we found no documents in the field of heart transplantation. There were (18/65) 28% of documents that did not determine a specific organ. Among this last category, (15/18) 83% were found using the keywords “non-heart-beating donor” in French or in English. Searching for “marginal donor”, (6/11) 55% and (7/10) 70% of the documents were dedicated, respectively, to liver transplant on websites in the French language and to kidney transplant on websites in the English language. Searching for “expanded criteria donor”, most of the documents were related to kidney transplant both in French and in English. No document was dedicated to heart transplantation.

Scientific articles (29/65), congress presentation (9/65), and protocol or report (6/65) constituted the specialized information intended for health care professionals. website pages (14/65) were the second main source of information. Other documents (7/65) included information leaflets, guidelines, lecture syllabus, and books.

Among 139 topics identified in documents, the information proposed was mainly about the definition of marginal donors (48/139, 34.5%), results of transplantation from marginal donors (34/139, 24.5%), the situation of organ shortages (18/139, 12.9%), and processes of marginal graft transplantation (15/139, 10.8%).

The only keywords that allowed finding information about ethical aspects were “non-heart-beating donor” in English or in French.

Among the Internet documents, (52/65) 80% were intended for health care professionals and (13/65) 20% for patients, knowing that the documents dedicated to the general population were counted as documents dedicated to patients. The sources of these last documents were different in each language. The French language documents were proposed by a French association (4/7), the ABM (1/7), a Belgian association (1/7), and a Swiss foundation (1/7). The English language documents were proposed by American hospitals (3/6), Wikipedia (1/6), and an American university (1/6).No English language documents intended for patients appeared in the Google search for “marginal donor”.

The documents published after 2009 were published more on French language websites (*P*=.001), dedicated to patients (*P*=.004), composed of general information (*P*=.003), and available to all Internet users (*P*=.024) than those published before.

### Third Survey

The global response rate of patient associations was (34/53) 64%. There were 10/34 kidney associations (29%), 9/34 liver (26%), 4/34 heart (12%), 1/34 lung (3%), 5/34 heart- lung (15%), and 1/34 association not dedicated to a specific organ (3%) that answered our questionnaire. There were 4/34 presidents (12%) who did not identify their own associations, preventing us from ascertaining the organ affected.

**Figure 1:**
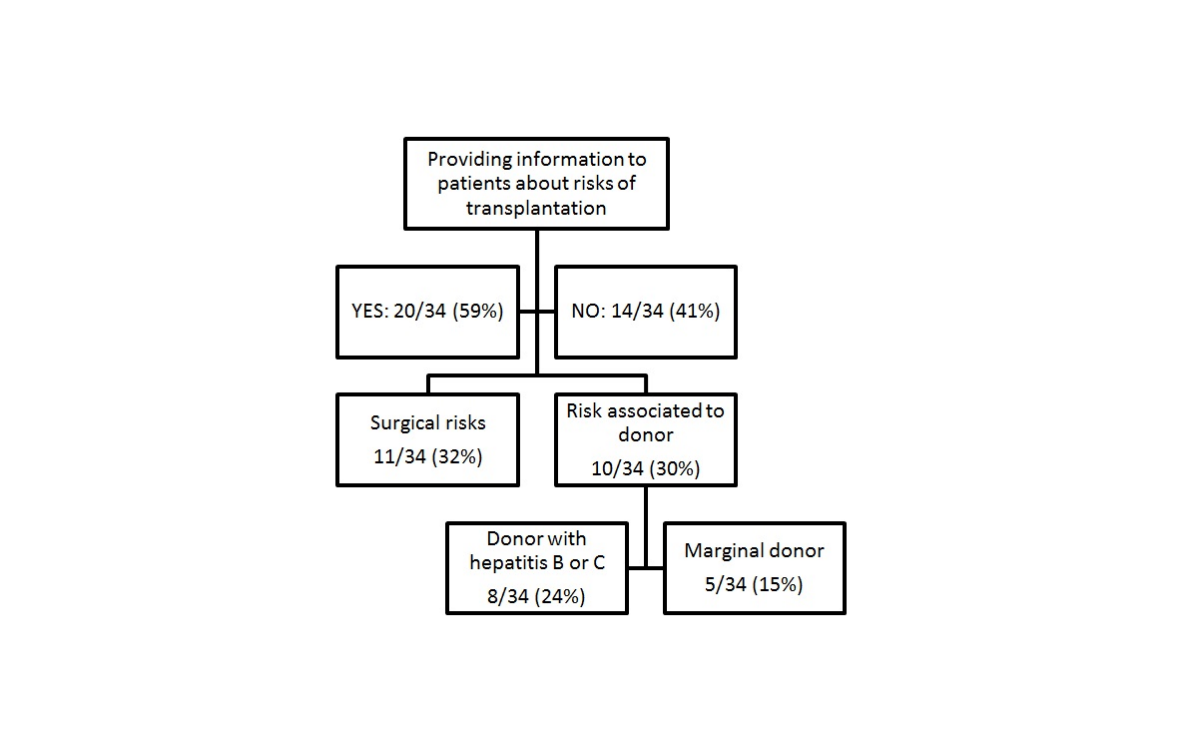
Flow chart of information proposed by associations to patients about the risks of transplantation.

There were (20/34) 59% of these associations that provided information to patients registered on the NWL about the risks of transplantation related to surgical procedures or to the risks associated to donors (ie, donor with hepatitis B or C, or marginal donor) or both (Figure 1 shows this).

The presidents of 12 associations did not answer the question asking for their ways to communicate with patients about the risks of transplantation. Among the 22 presidents who responded, (13/22) 59%, (8/22) 36%, (7/22) 32%, and (5/22) 23% of associations communicated with patients, respectively, by discussion groups involving patients that had already received transplants and patients registered on the NWL, patients’ meetings in hospital, written communication, and websites.

There were (24/32) 75% of the presidents who confirmed that transplanted patients and patients registered on the NWL could share experiences either by mentoring or by punctual meeting.

According to the responses of presidents, the patients often knew associations via other patients (23/30), leaflets available in the waiting rooms of outpatient consultations (21/30), physicians (20/30), and the associations’ websites (12/30).

## Discussion

### Principal Findings

Through one study including three surveys, we identified the information sources of transplant patients other than health care professionals, evaluated the information proposed by websites and patients associations about the risks of transplantation from marginal donors, and compared the information proposed by websites according to language, French or English. Patients’ knowledge about marginal donors was evaluated among kidney, liver, and heart transplant patients, and only a small proportion had heard about this type of donors.

For the first survey, 367/402 (91.3%) responses were registered. Apart from health professionals identified as the principal information source, 19 liver and 28 heart patients searched for information on the websites, while 37 kidney and 42 lung patients were informed by patients’ associations. Our two last surveys showed that information about marginal donors is accessible by websites and (10/34) 30% of associations. All the 60 Internet documents evaluated in websites in the French and English language proposed information about marginal donors. Otherwise, (52/65) 80% of these documents were dedicated to health professionals and contained specialized information difficult to understand by patients.

To our knowledge, the surveys focusing on health information sources [9,10] used the samples of the general population, but not patients. In our study, 338 patients had already received transplants and 29 patients on the NWL were questioned about their own information sources.

We focused on information rarely proposed by health care professionals to patients and tried to evaluate the accessibility of this type of information using other sources. Therefore, information proposed by other sources is considered as an alternative. Other studies [15-18] assessed the quality of information proposed to patients by other sources than health care professionals and the impact of this information on the physician-patient relationship. These studies suggested that information proposed by other sources was complementary to that proposed by health care professionals.

A survey among 3867 renal patients from 36 countries [19] found that health care professionals were more frequently scored as giving helpful information than patient organizations, websites, or social media. Our study also found that physicians and health care professionals were the most important information sources for transplant patients. Nevertheless, when an interested patient felt they were not being sufficiently informed, he or she could search the information somewhere else.

The source of Internet documents intended for patients was different in French and English language websites. Transplant centers offered half of the English language documents. While French patients’ associations prepared more than half of the French language documents intended for patients. Internet documents provided by health care professionals may be more trusted by patients than those provided by other sources. Our study found that physicians or other health care professionals informed all patients.

According to our three surveys, the ways in which the associations informed the patients about transplantation were not adapted to patients’ behavior. On the one hand, (45/115) 39.1% of patients considering an association as their main information source were informed by the association’s websites. However, only (5/22) 23% of the associations communicated the risks of transplantation with patients through their websites. On the other hand, more than half of the presidents of French patient associations stated that they mostly provided information to patients by “discussion groups involving already transplanted patients and patients registered on the NWL”. Communication tools proposed by these associations may not be well adapted to the patients’ demands. Nevertheless, only 15 patients considering associations as their main information source have already participated in a discussion group.

Accessibility of information proposed by other information sources than health care professionals depended on several factors: the knowledge of keywords by Internet users, the ability of searching in several languages, organ type and patient sociodemographic, and psychological characteristics.

The main information concerning marginal donors could be found by searching the specialized keywords. Searching simpler keywords, frequently used by patients (ie “transplantation” or “graft”), the risk communication was limited to “surgical risks” or “transplant rejection”. So, to access to useful information about marginal donors, patients should know that specialized keywords exist and know them.

Structured therapeutic education and using specialized keywords about marginal donors may facilitate Internet searches for US citizens. In the United States, therapeutic education programs [2,20-22] are organized for patients on the NWL and propose information about the risks of transplantation from marginal donors to patients. Moreover, the content of these programs are available on the websites of transplant centers. Therapeutic education about transplantation is not yet common in France. We found that only (51/367) 13.9% of French transplant patients have participated in a therapeutic education program. These programs are mostly organized for patients who had already received transplants and propose the information about post transplant care, particularly medication [23-28]. Additionally, the contents of these programs are not available on the websites. Therefore, bilingual patients who search for information both in English and in French on the websites may be more informed than others.

The type of organ is another factor influencing the type of information provided to patients. The Internet documents about marginal donors were often dedicated to kidney or liver transplantation, rarely to lung, and never to heart transplantation. In contrast, 44/51 patients (86.3%) participating in therapeutic education programs were heart or lung patients.

Only (47/280) 16.8% of kidney, liver, and heart patients have already heard about “marginal donors”. Physicians of transplant centers informed most of these patients.

The most important information sources apart from health care professionals were, for heart and liver patients, websites in the French language, and transplant associations for kidney and lung patients. Using the Internet as a source for heath information continues to increase. However, kidney and lung patients may have a special opportunity to be informed by patient associations. Kidney and lung associations are among the oldest associations of patients. They are devoted to patient information on their disease and their treatment since their beginning, before and after transplantation. Lung patients, particularly those suffering from cystic fibrosis, and kidney patients, during their dialysis, usually have contact with patient associations.

A minority of active patients, more able to understand medical topics than others, could be more informed than other patients thanks to reading the documents dedicated to health care professionals on websites, discussions with patients in the associations that had already received transplants, and searching for information in other languages on the Internet.

### Limitations

Our study had some limitations, especially concerning the first survey. First, most respondents had already received a transplant. This can be explained by the necessary recurrent outpatient visits for follow-up, while patients waiting for transplantation were supposed to come just once for pre transplant assessment. Second, the physicians of the lung transplant center chose to delete the question of our questionnaire concerning patients’ knowledge on marginal donors. Providing information to patients about marginal donors remains a taboo subject. Health care professionals, particularly lung and heart physicians, do not want to talk about this with patients. Furthermore, the physicians of kidney and liver transplant patients are not really much more prone to speak about this topic with their patients. Therefore, the transparency about marginal donors by health authorities could help to break down this taboo.

### Conclusions

Currently, patients want to be more informed by other information sources than health professionals, particularly by the websites. Patients could trust more websites if their physicians confirmed the reliability of information proposed by this source and informed patients about specialized keywords. Patients even expect physicians to recommend specific websites to them [29]. So the websites of universities, transplant centers, and associations should be improved also for dedicating the information for patients. It allows patients to have access to reliable information sources. Another conclusion of this study could be to improve the capacity of health professionals to communicate with patients, particularly by training the physicians in shared decision-making skills.

## Abbreviations

ABM: French National Agency of Transplantation (Agence de la Biomédecine)
NWL: national (French) waiting list
UNOS: United Network for Organ Sharing

## References

[1] (2013). Agence de la Biomédecine.

[2] (2008). United Network for Organ Sharing.

[3] (2013). Google AdWords.

[4] Bertrand X (2010). Journal Officiel de la République Française (JOFR).

[5] Antoine C, Tenaillon A (2007). Agence de la Biomédecine.

[6] Cormier M (2002). Actualité et Dossier en Santé Publique.

[7] Halpern SD, Shaked A, Hasz RD, Caplan AL (2008). Informing candidates for solid-organ transplantation about donor risk factors. N Engl J Med.

[8] Persson MO, Persson NH, Källén Ragnar, Ekberg H, Hermerén Göran (2002). Kidneys from marginal donors: Views of patients on informed consent. Nephrol Dial Transplant.

[9] Fox S, Duggan M (2013). Pew Internet.

[10] Vautrey A.S (2010). IPSOS.

[11] (2014). Agence de la Biomédecine.

[12] Laversine S (2007). Haute Autorité de Santé.

[13] Sullivan D (2013). Search Engine Land.

[14] (2013). Chitika.

[15] Givron P, Coudeyre E, Lopez S, Mares P, Hérisson C, Pelissier J (2004). [Quality assessment of information about female urinary incontinence from French speaking websites]. Ann Readapt Med Phys.

[16] Mathur S, Shanti N, Brkaric M, Sood V, Kubeck J, Paulino C, Merola AA (2005). Surfing for scoliosis: The quality of information available on the Internet. Spine (Phila Pa 1976).

[17] Lowrey W, Anderson WB (2006). The impact of internet use on the public perception of physicians: A perspective from the sociology of professions literature. Health Commun.

[18] Murray E, Lo B, Pollack L, Donelan K, Catania J, Lee K, Zapert K, Turner R (2003). The impact of health information on the Internet on health care and the physician-patient relationship: National U.S. survey among 1,050 U.S. physicians. J Med Internet Res.

[19] Van BW, Murphey M, Loblova O, Davies S, van der Veer Sabine N (2014). Patients' perceptions of information and education for renal replacement therapy: An independent survey by the European Kidney Patients' Federation on information and support on renal replacement therapy. PLoS One.

[20] (2011). Saint Barnabas Health Care System.

[21] (2012). Beth Israel Deaconess Medical Center- A Teaching Hospital of Harvard Medical School.

[22] (2010). University of California (UC DAVIS).

[23] (2011). infirmier.com.

[24] Hourmant M (2013). Centre Hospitalo Universitaire de Nantes.

[25] Pacaud E (2010). Université de Nantes.

[26] Pigneret-Bernanrd S (2008). Société de Néphrologie.

[27] (2013). Hospices civils de Lyon.

[28] (2011). Hôpital Pasteur.

[29] Diaz JA, Sciamanna CN, Evangelou E, Stamp MJ, Ferguson T (2005). Brief report: What types of Internet guidance do patients want from their physicians?. J Gen Intern Med.

